# A pseudotype baculovirus expressing the capsid protein of foot-and-mouth disease virus and a T-Cell immunogen shows enhanced immunogenicity in mice

**DOI:** 10.1186/1743-422X-8-77

**Published:** 2011-02-23

**Authors:** Yimei Cao, Zengjun Lu, Pu Sun, Yuanfang Fu, Feipeng Tian, Xiaofang Hao, Huifang Bao, Xiangtao Liu, Zaixin Liu

**Affiliations:** 1 Lanzhou Veterinary Research Institute of Chinese Academy of Agriculture Science, State Key Laboratory of Veterinary Etiological Biology, National Foot-and-Mouth Disease Reference Laboratory, Key Laboratory of Animal Virology of Ministry of Agriculture, Xujiaping No.1, Yanchangpu, Lanzhou, Gansu, 730046, PR China

## Abstract

**Background:**

Foot-and-mouth disease (FMD) is a highly contagious disease of livestock which causes severe economic loss in cloven-hoofed animals. Vaccination is still a major strategy in developing countries to control FMD. Currently, inactivated vaccine of FMDV has been used in many countries with limited success and safety concerns. Development of a novel effective vaccine is must.

**Methods:**

In the present study, two recombinant pseudotype baculoviruses, one expressing the capsid of foot-and-mouth disease virus (FMDV) under the control of a cytomegalovirus immediate early enhancer/promoter (CMV-IE), and the other the caspid plus a T-cell immunogen coding region under a CAG promoter were constructed, and their expression was characterized in mammalian cells. In addition, their immunogenicity in a mouse model was investigated. The humoral and cell-mediated immune responses induced by pseudotype baculovirus were compared with those of inactivated vaccine.

**Results:**

Indirect immunofluorescence assay (IFA) and indirect sandwich-ELISA (IS-ELISA) showed both recombinant baculoviruses (with or without T-cell epitopes) were transduced efficiently and expressed target proteins in BHK-21 cells. In mice, intramuscular inoculation of recombinants with 1 × 10^9 ^or 1 × 10^10 ^PFU/mouse induced the production of FMDV-specific neutralizing antibodies and gamma interferon (IFN-γ). Furthermore, recombinant baculovirus with T-cell epitopes had better immunogenicity than the recombinant without T-cell epitopes as demonstrated by significantly enhanced IFN-γ production (*P *< 0.01) and higher neutralizing antibody titer (*P *< 0.05). Although the inactivated vaccine produced the highest titer of neutralizing antibodies, a lower IFN-γ expression was observed compared to the two recombinant pseudotype baculoviruses.

**Conclusions:**

These results indicate that pseudotype baculovirus-mediated gene delivery could be a alternative strategy to develop a new generation of vaccines against FMDV infection.

## Background

Foot-and-mouth disease (FMD) is a highly contagious disease of cloven-hoofed animals. The causative agent is foot-and-mouth disease virus (FMDV) which belongs to the genus *Aphthovirus *in the family *Picornaviridae *[1]. Foot-and-mouth disease is a major hindrance to international trade in animals and animal products. Prevention and eradication of this disease in one country requires sustained effort at significant cost. Vaccination is still a major strategy in developing countries to control FMD. Current FMDV vaccines are available in the form of BEI inactivated antigen in oil adjuvant or aluminum hydroxide and saponin adjuvant [2]. Although these vaccines can induce humoral protective immunity, there are a number of disadvantages with their use, including the inability to differentiate vaccinated from unvaccinated animals, the short-term nature of protection, the extra cost of containment facilities required for their preparation, and the risk of escaped virus [3,4]. Thus, it is crucial to develop alternative vaccines.

Since Hofmann reported that recombinant baculovirus containing the cytomegalovirus immediate-early promoter (CMV-IE) was able to drive the expression of a reporter gene in human hepatocytes, baculovirus with a strong mammalian promoter has been used as a novel vector to transfer and express foreign genes in mammalian cells for vaccine development [5-7]. This vector was also shown to be capable of carrying large inserts and infecting a variety of cell lines without any apparent viral replication or cytopathic effects, even at a high multiplicity of infection (MOI) [7,8]. Furthermore, it has been reported that a pseudotype baculovirus displaying the glycoprotein of vesicular stomatitis virus (VSV-G) on the envelope can extend the host range, increase the transduction efficiency, and prolong the baculovirus-mediated expression in mammalian cells [9,10].

The use of baculovirus as a vector for vaccination was initially described by Aoki and coworkers, who demonstrated that injecting mice with a recombinant vector expressing pseudorabies virus glycoprotein B elicited a measurable humoral response directed against this viral glycoprotein [11]. More recently, direct vaccination with recombinant pseudotype baculovirus induced high-level humoral and cell-mediated immunity against various antigens such as influenza virus HA [12], porcine reproductive and respiratory syndrome virus (PRRSV) [13], Japanese encephalitis virus (JEV) [14], porcine circovirus type 2 (PCV2) [15], *Toxoplasma gondii *[16], and *Plasmodium falciparum *[17].

Although it is generally accepted that protective immunity to FMDV is principally due to a neutralizing antibody, a T-cell response is quite clearly necessary for effective immunity; this was demonstrated in pigs that showed no consistent humoral immune response after inoculation with inactivated vaccine but could still resist virulent virus challenge. It is now believed that cell-mediated immunity is crucial for protection against FMD. Helper T (Th) lymphocyte epitopes with conserved sequences among different FMDV isolates, and that are recognized by a wide spectrum of MHC Class II alleles in different host species, hold great potential for vaccine design. Residues 20-34 in the structural protein VP4 [18,19] and T-cell epitopes identified on the FMDV non-structural proteins 3D [20,21] and 3A [18] are highly interspecies MHC-restricted Th lymphocyte epitopes. Such epitopes have the additional advantage of being recognized in a heterotypic manner by T-cells of different individuals. The potential of such Th epitopes to improve immunogenicity of a new FMDV vaccine is an ongoing focus of investigation.

Based on these observations, a T-cell epitope fragment was designed with two universal T-cell epitopes and several conservative T-cell epitopes on the FMDV structural and non-structural proteins. Two recombinant pseudotype baculoviruses encoding P12A and 3C, with or without insertion of the above T-cell epitopes, were constructed and their expression was characterized in mammalian cells. In addition, their immunogenicity in a mouse model was investigated. The humoral and cell-mediated immune responses induced by pseudotype baculovirus were compared with those of inactivated vaccine. The results obtained further demonstrate that significant cell-mediated immunity to target antigens can be elicited upon injection of recombinant pseudotype baculovirus.

## Materials and methods

### Virus and cultured cells

O-serotype FMDV (strain O/HN/CHA/93 with high homology to O/GD/CHA/86, GenBank AJ131468) was provided by the National Foot-and-Mouth Disease Reference Laboratory of China to clone the P1-2A and 3C genes of O type FMDV. The BHK-21 cells were grown and maintained in Dulbecco's modified Eagle's medium (DMEM, Invitrogen) supplemented with 10% heat-inactivated fetal bovine serum (FBS). *Spodoptera frugiperda *9 (Sf-9) insect cells were maintained at 27°C in Sf-900 II SFM (Invitrogen) supplemented with 2.5% fetal bovine serum.

### Construction of recombinant pseudotype baculovirus

**Figure 1:**
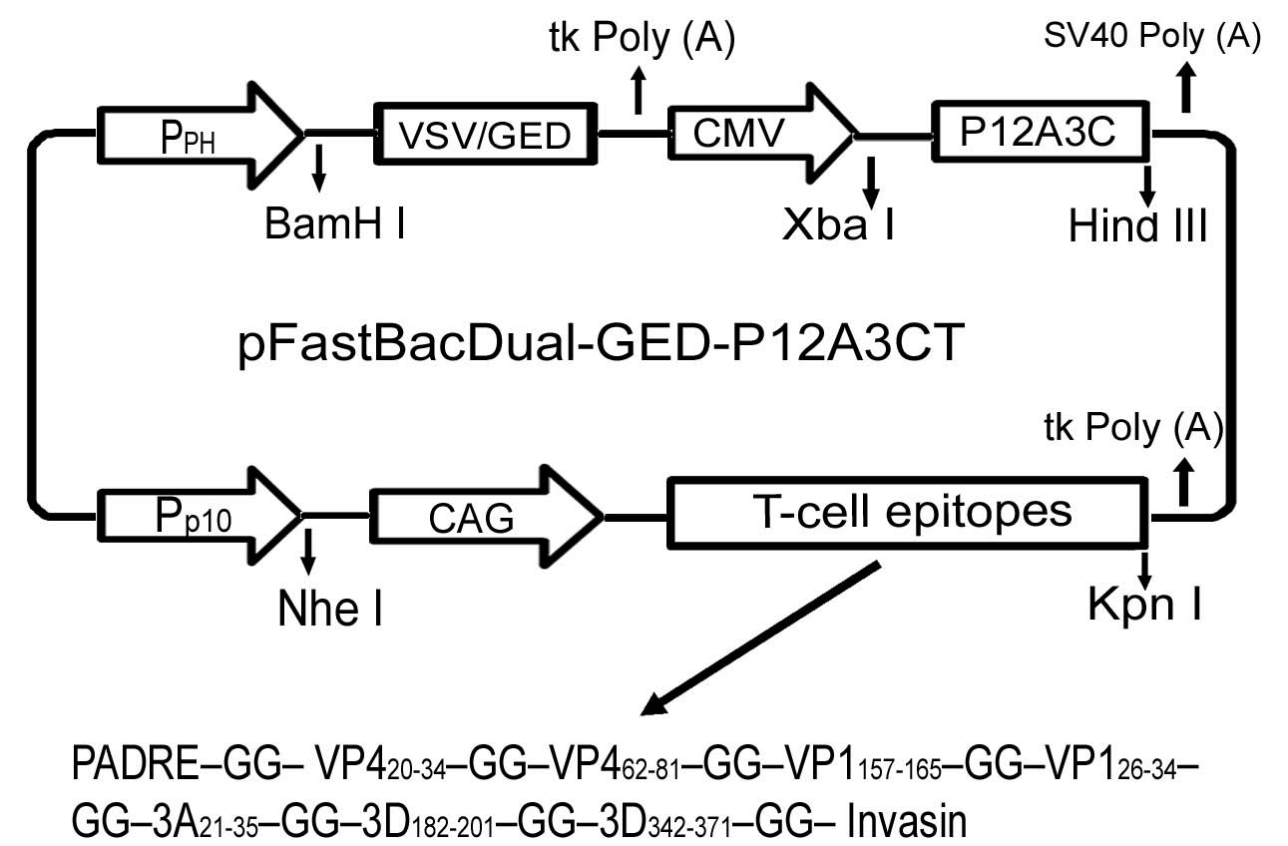
**Schematic diagram of the transfer plasmid for construction of recombinant baculovirus**. The structure of the transfer plasmid based on pFastBac Dual is displayed. pH, polyhedrin promoter of baculovirus; p10, p10 promoter of baculovirus; VSV/GED, truncated glycoprotein of vesicular stomatitis virus including 21 amino acid ectodomain with transmembrane and cytoplasmic tail domains; CMV, cytomegalovirus immediate-early promoter/enhancer; SV40 or tk poly (A), polyadenylation signal from Simian virus 40 or tk gene of Herpes simplex virus; P12A3C, the P12A3C gene encoding structural and non-structural proteins necessary for the formation of FMDV empty capsid; CAG, a composite promoter consisting of the CMV IE enhancer and chicken β-actin promoter; T-cell epitopes including a pan DR epitope sequence (PADRE), seven T cell epitope regions in structural or non-structural proteins of FMDV and a invasin immunostimulatory sequence taken from Yersinia, as described by Wang et al. (2002).

In order to construct a pseudotype baculovirus, a DNA fragment was synthesized that contained a coding region of a truncated VSV-G, a herpes simplex virus-thymidine kinase (HSV tk) Poly(A) terminal sequence, and a CMV-IE promoter flanked with *Bam*H I and *Xba *I restriction sites. The truncated VSV-G includes a 21 amino acid ectodomain with transmembrane and cytoplasmic tail domains (VSV-GED) as described by Kaikkonen et al. [22]. The above DNA fragment was synthesized by GenScript Corporation (Centennial Ave., Piscataway, NJ 08854, U.S.) with codon optimization for expression in insect cells. This DNA fragment was then inserted into pFastBac Dual vector (Invitrogen) under the control of the polyhedron (PH) promoter via *Bam*H I and *Xba *I restriction sites. This vector was designated pFastBacDual-GED. The DNA fragment containing the P12A3C expression cassette of O-type FMDV was inserted into pFastBacDual-GED via *Xba *I and *Hind *III restriction sites, resulting in the recombinant transfer plasmid pFastBacDual-GED-P12A3C (Figure 1).

To express a T-cell epitope region in mammalian cells, we utilized the CAG promoter, a composite promoter consisting of the CMV IE enhancer and chicken β-actin promoter [7]. The CAG promoter (737 bp) and a T-cell epitope region (495 bp) were synthesized by GenScript Corporation and inserted into pFastBacDual-GED-P12A3C downstream of the p10 promoter followed by the HSV tk Poly (A) tail. The result was the recombinant transfer plasmid pFastBacDual-GED-P12A3CT. The T-cell epitopes synthesized contained two universal immunostimulatory elements and several T-cell epitopes on structural and non-structural proteins of FMDV (Table 1).

**Table 1:** T-cell epitopes included in the synthesized T-cell immunogen

Epitopes	Sequence	Function
PADRE	AKFVAAWTLKAAA	T_H_
VP4_20-34_	SIINNYYMQQYQNSM	T_H_
VP4 _62-81_	TQNNDWFSKLASSAFSGLFG	T_H_
VP1_157-165_	RTLPTSFNY	Tc
VP1_26-34_	RRQHTDVSF	Tc
3A_21-35_	AAIEFFEGMVHDSIK	T_H_
3D_182-201_	VDVLPVEHILYTRMMIGRFC	T_H_
3D_342-371_	VVASDYDLDFEALKPHFKSLGQTITPADKS	T_H_
Invasin	TAKSKKFPSYTATYQF	T_H_

The T-cell epitopes were ligated sequentially via two glycines (Gs) as following: PADRE-GG-VP4_20-34_-GG-VP4 _62-81_-GG-VP1_157-165_-GG-VP1_26-34_-GG-3A_21-35_-GG-3D_182-201_-GG-3D_342-371_-GG- Invasin

The recombinant baculoviruses Bac-GED, Bac-GED-P12A3C, and Bac-GED-P12A3CT were subsequently generated using the Bac-to-Bac System (Invitrogen). Briefly, the recombinant plasmids were transformed into *Escherichia coli *DH10Bac (Invitrogen), in which all the expression cassettes between Tn7R and Tn7L had been transferred from pFastBacDual-GED, pFastBacDual-GED-P12A3C, and pFastBacDual-GED-P12A3CT to the bacmid by site-specific transposition. The subsequent steps for bacmid isolation, transfection, and selection of the recombinant viruses were performed according to the manufacturer's instructions for the Bac-to-Bac System. Recombinant baculoviruses were amplified further by propagation in Sf-9 cells. Virus purification was performed as described previously [23] and purified virus was resuspended in phosphate-buffered saline (PBS, pH 7.4). The virus titer was determined by the BacPAK Rapid Titer assay (Clontech, Mountain View, CA, USA) in Sf-9 cells.

### Baculovirus transduction and protein expression assay

The BHK-21 cells were seeded at a concentration of 2.5 × 10^5 ^cells/well into six-well tissue culture plates (Nunc) until the cells reached approximately 70-80% confluence. Culture medium was removed and cells were washed three times with PBS (pH 7.4). The cells were then incubated in media containing baculovirus for 6 h at 27°C. After removal of virus, fresh medium was added and cultures were incubated at 37°C. At 48 h post-transduction, cells were analyzed for expression of FMDV proteins by the indirect immunofluorescence assay (IFA) and indirect sandwich-ELISA (IS-ELISA). Briefly, cells were fixed with 4% paraformaldehyde and processed for indirect immunofluorescence assay (IFA) using rabbit serum against FMDV 146S antigen, followed by fluorescein isothiocyanate-conjugated goat anti-rabbit IgG. The unbound fluorescent antibodies were washed away with PBST and the cells were sealed with glycerol and observed under a fluorescence microscope. Indirect sandwich-ELISA was performed as described previously [24]. Expression of the VSV-GED protein was monitored in Sf-9 cells by IFA with a rabbit monoclonal antibody specific for VSV-G (Sigma), and the recombinant baculovirus Bac-P12A3C constructed previously was used as a mock-infected control [24].

### Immunization of mice

Six to eight weeks old female BALB/c mice, purchased from the Animal Center, Lanzhou Institute of Biological Products, Gansu Province, 730046, China, were randomly divided into seven groups with eight mice per group. Two groups were injected intramuscularly with 1 × 10^9 ^PFU and 1 × 10^10 ^PFU of Bac-GED-P12A3C. Two groups were injected intramuscularly with 1 × 10^9 ^and 1 × 10^10 ^PFU of Bac-GED-P12A3CT. The other three groups were immunized intramuscularly with 1 × 10^10 ^PFU of Bac-GED, 100 μL of PBS, or 100 μL inactivated vaccine. Booster immunization was performed identically 3 weeks later. Serum samples were collected from the retro-orbital plexus at 3 and 7 weeks after immunization for serological tests. At 7 weeks after the primary immunization, mice were sacrificed and splenocytes were harvested for IFN-γ assay. All animal studies were approved by the Review Board of Lanzhou Veterinary Research Institute, Chinese Academy of Agricultural Sciences (Permission number: SYXK-GAN-2004-0005). The mice in this work were kindly bred during the experiment and mercy-killed at the end of the experiments to reduce the suffering at the most extent.

### Detection of specific neutralizing antibodies against FMDV

Serum samples were heat-inactivated for 45 min at 56°C before testing. All sera were analyzed for neutralizing antibody titers by using a micro-neutralization assay with monolayers of BHK-21 cells [24]. Double dilutions of sera were reacted with 100 TCID_50 _of FMDV O/HN/CHA/93 at 37°C for 1 h. Cells were then added as indicators of residual infectivity. The microplates were incubated at 37°C for 3 days prior to fixation and staining. The endpoint titers were calculated as the reciprocal of the last serum dilution to neutralize 100 TCID_50 _homologous FMDV in 50% of the wells.

### IFN-γ release assay

Mouse splenocytes were isolated as described previously [25]. Splenocytes (1 × 10^6^/ml) were cultured in 24-well plates at 37°C in the presence of 5% CO_2_, in the presence or absence of 20 μg/ml FMDV 146S antigen. After 20 h incubation, culture supernatant was harvested and the presence of IFN-γ was tested using a commercial mouse IFN-γ immunoassay ELISA kit (BD Bioscience) according to the manufacturer's instructions. The concentrations of IFN-γ in the samples were determined from the standard curves.

### Statistical analysis

Student's t-test was used to compare the humoral and cellular immune responses between the different groups. *P*-values of < 0.05 were considered statistically significant.

## Results

### Construction and identification of recombinant transfer plasmid

A schematic diagram of the transfer plasmid for construction of recombinant pseudotype baculovirus is illustrated in Figure 1. The recombinant transfer plasmids pFastBacDual-GED-P12A3C and pFastBacDual-GED-P12A3CT were confirmed by restriction digestion, PCR, and sequence analysis, and there were no mutations introduced in target genes.

For construction of the T-cell immunogen, two universal T-cell epitopes were chosen, a pan HLA DR-binding peptide (PADRE) [26] and an invasin immunostimulatory sequence taken from *Yersinia *(Invasin) [27]. Eight conservative T-cell epitopes within the structural and non-structural proteins of O/HN/CHA/93 FMDV [28] were linked in tandem by two glycine (G) residues according to the order in Table 1. The DNA sequence of the designed T-cell immunogen (as shown in Table 1) was synthesized by GenScript Incorporation (http://www.genscript.com) according to the most commonly occurring codons in mammalian cells.

### Expression of the target proteins by recombinant baculoviruses in mammalian cells

**Figure 2:**
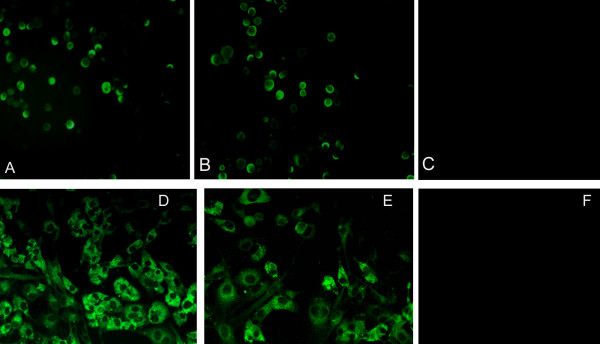
**Detection of the VSV-G protein in baculovirus-infected Sf9 cells (A-C) and FMDV capsid proteins in BHK-21 cells (D-F) by immunofluorescence**. VSV-G proteins were detected in Sf9 cells infected with baculovirus Bac-GED-P12A3C (A), Bac-GED-P12A3CT (B), or Bac-P12A3C (C) by staining with a monoclonal antibody against VSV-G. FMDV capsid proteins were detected in BHK-21 cells after infection with baculoviruses Bac-GED-P12A3C (D) and Bac-GED-P12A3CT (E) at 48 h post-infection by reaction with a rabbit serum against FMDV 146S antigen. No positive reaction was shown in BHK cells transduced with Bac-GED (F).

To determine whether Bac-GED-P12A3C and Bac-GED-P12A3CT contain and express the VSV-GED protein, purified virus was analyzed by IFA with a VSV-GED-specific monoclonal antibody. Expression of the VSV-GED protein was detected both in the Bac-GED-P12A3C (Figure 2A) and Bac-GED-P12A3CT (Figure 2B) preparation, whereas no VSV-G protein was present in the Bac-P12A3C viral preparation (Figure 2C).

**Figure 3:**
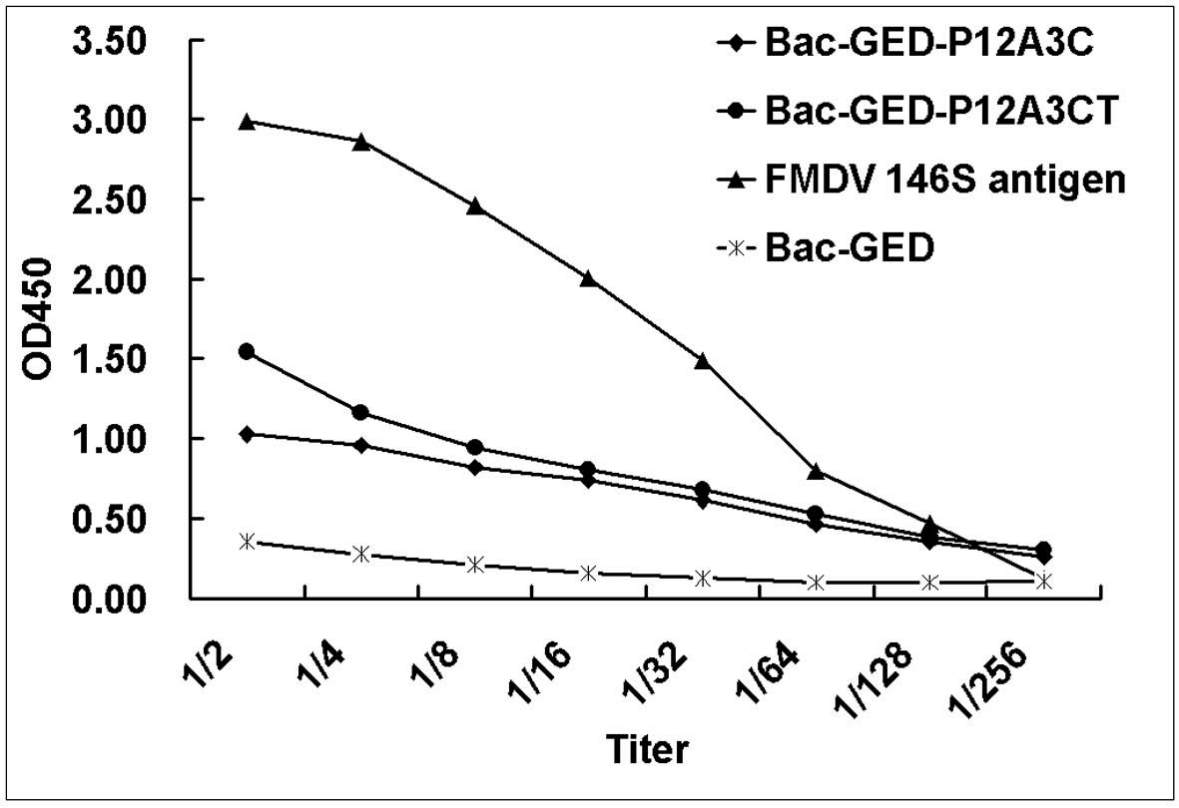
**Detection of protein expression in baculovirus-transduced BHK cells by IS-ELISA**. Cell lysates from BHK cells transduced with baculovirus were prepared at 48 h post-transduction and diluted by two-fold serial dilution. The data are presented as the mean of OD450 for each dilution.

To investigate the transduction efficacy of recombinant baculoviruses and the level of FMDV capsid proteins expression within mammalian cells, BHK-21 cells were transduced with Bac-GED-P12A3C or Bac-GED-P12A3CT at a multiplicity of infection (MOI) of 100. An indirect immunofluorescence assay and IS-ELISA were performed at 48 h post-transduction to detect the FMDV capsid proteins expressed in BHK-21 cells. As shown in Figure 2D-F, cells transfected with Bac-GED-P12A3C (Figure 2D) and Bac-GED-P12A3CT (Figure 2E) emitted bright fluorescence, but no detectable fluorescence was observed from cells transduced with Bac-GED (Figure 2F). Furthermore, the OD of the harvested cell lysates transfected with Bac-GED-P12A3C and Bac-GED-P12A3CT decreased with greater dilution, but no changes were observed in the BHK-21 cell lysates transfected with Bac-GED (Figure 3).

### FMDV-specific neutralizing antibodies elicited by Bac-GED-P12A3C and Bac-GED-P12A3CT in mice

**Figure 4:**
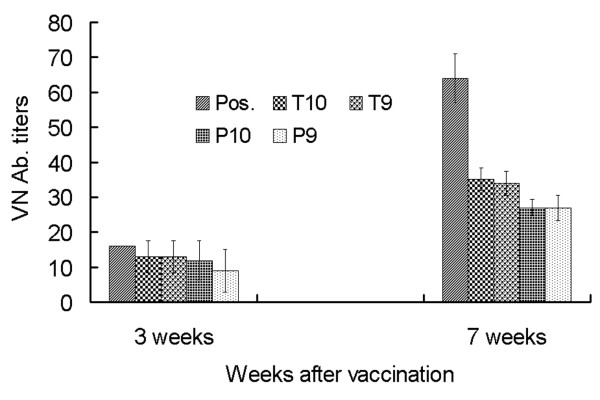
**Neutralizing antibody titers in immunized mice. Neutralizing antibody determinations were performed with sera sampled at 3 and 7 weeks after the primary vaccination**. Serum samples were obtained from five mice from each group. Data represent the mean ± SD. P9, P10 show mice vaccinated with 1 × 10^9 ^or 1 × 10^10 ^PFU of Bac-GED-P12A3C; T9, T10 show mice vaccinated with 1 × 10^9 ^or 1 × 10^10 ^PFU of Bac-GED-P12A3CT; "inactivated" denotes mice vaccinated with 100 μL inactivated vaccine.

To determine whether the pseudotype baculovirus expressed proteins could induce FMDV-specific humoral immune responses *in vivo*, BALB/c mice were immunized intramuscularly with two dosages (1 × 10^10 ^and 1 × 10^9 ^PFU/mouse) of Bac-GED-P12A3C and Bac-GED-P12A3CT, while PBS, Bac-GED, and inactivated vaccine were used as controls. Foot-and-mouth disease-specific neutralizing antibodies were monitored at 3 and 7 weeks after the primary immunizations. As shown in Figure 4, mice immunized with 1 × 10^10 ^and 1 × 10^9 ^PFU of Bac-GED-P12A3C developed mean neutralizing antibody titers of 1:12 and 1:9 at 3 weeks, respectively, and both increased to 1:27 at 7 weeks after primary inoculation. Mice immunized with 1 × 10^10 ^and 1 × 10^9 ^PFU of Bac-GED-P12A3CT produced mean neutralizing antibody titers of 1:13 at 3 weeks which further increased to 1:35 and 1:34 at 7 weeks after primary inoculation. This was significantly higher than Bac-GED-P12A3C injected mice (*n = 5, P < 0.05*). As expected, sera from mice immunized with Bac-GED or PBS did not display any neutralizing antibody activity throughout the entire duration of study. However, the inactivated vaccine group exhibited a much higher neutralizing antibody titer than any other group (*n = 5, P < 0.01*), reaching 1:16 at 3 weeks and 1:64 7 weeks after primary inoculation (Figure 4).

### Cellular immunity elicited by recombinant pseudotype baculoviruses in mice

**Figure 5:**
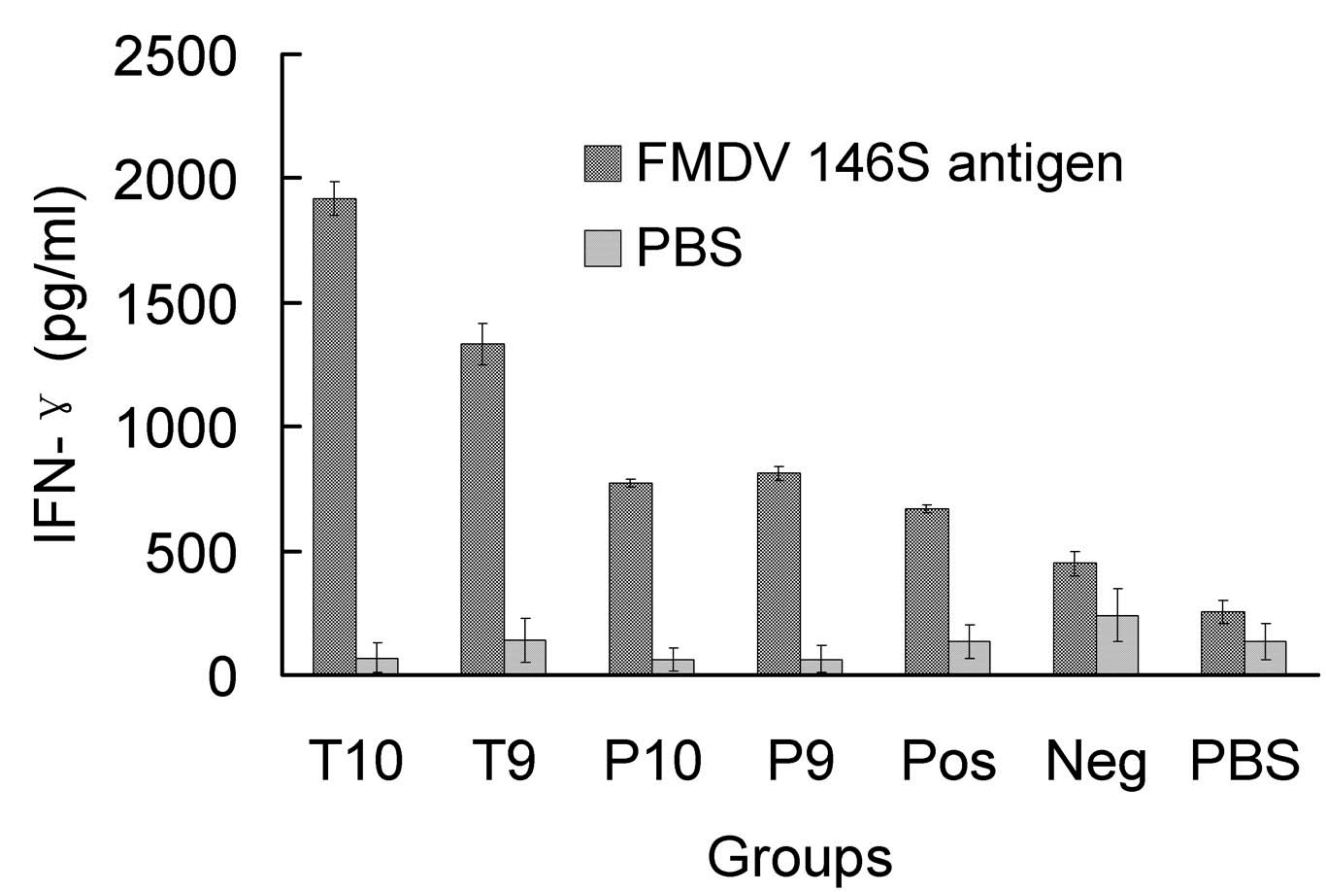
**IFN-γ production in the supernatant of splenocytes harvested from immunized mice after in vitro restimulation**. Mice were immunized as in Figure 4. Splenocytes were isolated 7 weeks after primary immunization and were restimulated in vitro with 146S antigen of FMDV for 20 h. IFN-γ production in the supernatant was analyzed by ELISA. Data represent the mean ± SD. Pos, Neg, or PBS denotes mice vaccinated with 100 μL inactivated vaccine, 1 × 10^10 ^PFU of Bac-GED, or 100 μL of PBS.

To further characterize the cell-mediated immune responses in mice immunized with Bac-GED-P12A3C and Bac-GED-P12A3CT, mice were killed at 7 weeks after primary immunization and IFN-γ production in splenocytes was measured by ELISA after restimulation with FMDV 146S antigen. As shown in Figure 5, mean IFN-γ production reached 1917 and 1332 pg/ml in mice inoculated with 1 × 10^10 ^and 1 × 10^9 ^PFU of Bac-GED-P12A3CT, which was significantly higher than splenocytes from mice that received the same two doses of Bac-GED-P12A3C (771 and 813 pg/ml, respectively) or inactivated vaccine (667 pg/ml) (*n = 3*, *P *< 0.01). As expected, no significant production of IFN-γ was detected in PBS-inoculated mice. But splenocytes harvested from baculovirus Bac-GED injected mice produced a relatively higher background non-specific IFN-γ response (450 pg/ml).

## Discussion

Development of a novel vaccine against FMD will have a significant impact on livestock farming and trade. A superior immunization protocol should possess improved immune duration, greater cross protection between different serotypes and genotypes of FMDV, and facilitate the differentiation of vaccinated from infected animals. Recent advances in immunology and molecular biology have stimulated the development of gene-based vaccines. The first is based on the use of plasmid DNA encoding the target antigen of FMDV [29]. However, the use of plasmid DNA as a gene transfer vehicle for immunization is somewhat limited by the low transduction efficiency that hampers its immunogenic potential. To overcome this problem, live virus vector-based vaccine is one possible strategy. Recombinant vaccinia virus and adenovirus for expression of capsid protein has been reported previously [30-32]. Only recombinant adenovirus expressing the empty capsid of FMDV could elicit protective immunity in the host [33-35]. Swine vaccinated with recombinant adenovirus could be protected from virulent virus challenge even with low titers of neutralizing antibody [36]. In the study by Sanz-Parra et al. [32], immunized pigs could develop protective immunity by FMDV-specific T-cell responses but had no detectable neutralizing antibodies. These studies indicated lack of neutralizing antibodies does not necessarily mean absence of protection, at least in pigs, despite the common belief that humoral immunity against FMD is the most important factor determining the efficiency of protection. Thus, the protection observed in these studies was likely mediated by the cellular immune response. In addition, some conserved T-cell epitopes in VP4 and 3A were shown to induce specific immune responses mediated by CD4^+ ^T lymphocytes and B lymphocytes, and the higher Th cell response could enhance the production of anti-FMDV neutralizing antibodies [18,37].

Based on these observations, two pseudotype baculoviruses, Bac-GED-P12A3C and Bac-GED-P12A3CT, were constructed to express the empty capsid and a T-cell immunogen with the expectation that such construction could elicit high levels of anti-FMDV neutralizing antibodies. Among these genes, the P1 sequence contains important B-lymphocyte and T-lymphocyte epitopes, allowing stimulation of the cellular and humoral immune responses, and 3C is a protease necessary for processing of the P12A polyprotein into VP1, VP3 and VP0, the components of self-assembling empty capsids. The T-cell immunogen was designed with two universal T-cell epitopes and several specific T-cell epitopes in the structural and non-structural proteins of FMDV to further enhance the immunogenicity of recombinant pseudotype baculovirus. Both the humoral and cellular immune responses were investigated in a mouse model vaccinated with pseudotype baculoviruses. All mice immunized with various dosages of Bac-GED-P12A3C and Bac-GED-P12A3CT developed detectable neutralizing antibodies, but Bac-GED-P12A3CT infection resulted in a higher antibody response. This indicated that the involvement of the T-cell immunogen could enhance the production of anti-FMDV neutralizing antibodies. However, mice inoculated with inactivated vaccine produced much higher titers of neutralizing antibodies than Bac-GED-P12A3C and Bac-GED-P12A3CT, indicating that the amount of capsid protein expressed by pseudotype baculoviruses did not reach that in the inactivated vaccine.

In addition to the humoral immune response, cell-mediated immunity has also been suggested to confer protective immunity against FMDV [32,38,39]. Host IFN-γ production plays a critical role in directing the cell-mediated immune responses for the clearance of intracellular pathogens [40]. Therefore, the production of IFN-γ was measured to evaluate the immunogenicity of Bac-GED-P12A3C and Bac-GED-P12A3CT in mice. All mice immunized with various dosages of Bac-GED-P12A3CT produced much higher levels of IFN-γ than mice infected with Bac-GED-P12A3C, indicating that insertion of a T-cell immunogen in Bac-GED-P12A3CT baculovirus could induce a greater cellular immune responses than Bac-GED-P12A3C. Although inactivated vaccine could elicit more neutralizing antibody than either Bac-GED-P12A3C or Bac-GED-P12A3CT, the IFN-γ test demonstrated lower levels of cellular immunity. Indeed, mice immunized with Bac-GED-P12A3C and Bac-GED-P12A3CT produced significantly higher levels of IFN-γ compared with mice that received inactivated vaccine. One possible reason was that baculovirus vectors can not only transduce mouse skeletal muscle cells, but also efficiently transduce dendritic cells (DCs), the most important APCs [17]. The "adjuvant" effect of baculovirus should also be considered. Baculovirus has the ability to induce innate immune responses through the Toll-like receptor 9 dependent signaling pathway, resulting in the production of various cytokines, including tumor necrosis factor-α, IL-6, and interferon [12,23,41,42]. In this study, a higher background of non-specific IFN-γ production was detected from mice injected with Bac-GED recombinant baculovirus, which further suggested the presence of the "adjuvant" effect of baculovirus. A similar result has been observed in pseudotype baculovirus expressing PRRSV's GP5 and M protein [13], JEV's E protein [14], PCV2's ORF2 protein [15], and *Toxoplasma gondii *SAG1 protein [16].

## Conclusion

In conclusion, Bac-GED-P12A3CT that expressed a T-cell immunogen and FMDV capsid protein had superior immunogenicity to Bac-GED-P12A3C that expressed only the capsid proteins of FMDV, as demonstrated by enhanced IFN-γ production and neutralizing antibody titer in mice. The combination of caspid protein and T-cell epitopes in Bac-GED-P12A3CT recombinant pseudotype baculovirus could be a promising strategy for the development of a new generation of vaccines against FMDV.

## Competing interests

The authors declare that they have no competing interests.

## Authors' contributions

YMC performed most of the experimental work and drafted the manuscript. PS, FPT and XFH participated in the immunization of mice. YFF helped with the ELISA assay. HFB and XTL participated in the analysis of humoral and cellular responses. ZJL and ZXL designed the study, revised the manuscript for important intellectual content and gave final approval of the version to be published. All authors read and approved the final manuscript.
